# Supplementary material for: Reshaped three-body interactions and the observation of an Efimov state in the continuum
Source: Nat Commun. 2024 Mar 8;15:2127. doi: 10.1038/s41467-024-46353-1 (PMC10923905; doi:10.1038/s41467-024-46353-1)
Supplement: Supplementary file 1 — Supplementary Information [file 41467_2024_46353_MOESM1_ESM.pdf]

SUPPLEMENTARY INFORMATION
Reshaped Three-Body Interactions and the Observation of an Efimov State in the Continuum

Yaakov Yudkin[1], Roy Elbaz[1], José P. D'Incao[2,3], Paul S. Julienne[4], and Lev Khaykovich[1]
[1]*Department of Physics, QUEST Center and Institute of Nanotechnology and Advanced Materials, Bar-Ilan University, Ramat-Gan 5290002, Israel*
[2]*JILA, University of Colorado and NIST, Boulder, Colorado 80309-0440, USA*
[3]*Department of Physics, University of Colorado, Boulder, Colorado 80309-0440, USA and*
[4]*Joint Quantum Institute (JQI), University of Maryland and NIST, College Park, Maryland 20742, USA*
(Dated: February 19, 2024)

Supplementary Note 1. MAGNETIC MOMENT

The fact that the trimer's nature changes after the crossing is most dominantly shown by looking at the magnetic moment of the trimer before and after the crossing. This is extracted from our data as follows. The slope of a molecules' binding energy, when plotted as a function of the magnetic field, is given by the magnetic moment of the molecule with respect to the free atoms. Since our Feshbach resonance is located at high magnetic fields the electronic spins of the free atoms are almost perfectly polarized (almost $2\mu_B$, where $\mu_B = 1.4$ MHz/G is the Bohr magneton). The magnetic moment of two free atoms for the relevant magnetic fields is $\mu_{AA} = 2.66$ MHz/G while the deeply bound dimer is a pure singlet with zero magnetic moment. The dimer is relatively shallow in this regime but deep enough to show non-universality. We extract its magnetic moment μ_D by performing a derivative of its binding energy E_D: $\mu_D = \mu_{AA} - \partial E_D/\partial B$. Adding the third atom as a free atom (moment $\mu_A = 1.33$ MHz/G) the polarized, three particle, dimer + atom system has $\mu_{DA} = \mu_D + \mu_A$. This is plotted as a function of the inverse scattering length in Supplementary Fig. 1. The magnetic moment of the trimer is $\mu_T = 3\mu_A - \partial E_T/\partial B$. Applying a discrete derivative to our measurement of E_T before it vanishes below the lower detection limit (before the crossing) results in the brown circles in Supplementary Fig. 1. The almost-overlap with μ_{DA} indicates that the trimer is very similar in nature to the dimer + atom system. However, the slope after crossing (see linear fit in Fig. 3 of the main text, the slope is ∓ 171 kHz/G) has a larger magnitude leading to a distinct change in μ_T (blue dot in Supplementary Fig. 1). This change in the magnetic moment provides an additional evidence of the emergence of the trimer as a bound state above the atom-dimer continuum. If μ_T remained unchanged, it would instead indicate the dissociation of the trimer state. We conclude that the nature of the trimer state changes as it emerges from below of the detection limit as a result of the reshape of the three-body interactions leading to the formation of the repulsive barrier near $R \approx 4r_{\rm vdW}$ (see Fig. 4 of the main text).

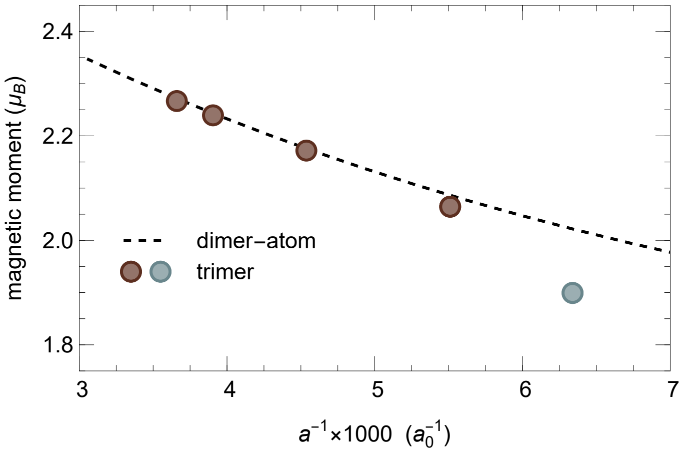

Supplementary Fig. 1. **Magnetic moment.** The magnetic moment of the measured trimer is compared to the dimer + atom complex. The scattering length calibration is discussed in Methods and the errorbars (1σ) are smaller than the point size in both the horizontal and vertical direction.

Supplementary Note 2. DATA ANALYSIS

Here we elaborate on the idea and procedure of the three-parameter fit analysis (3PA) used to extract the dominant frequency contribution from our low-SNR data. Please also refer to the Supplement of Ref. [1], where this method was first introduced.

A. Detailed description of the three-parameter analysis

In order to illustrate our data analysis it is instructive to apply it to a simulated data sequence. Consider a finite-length sinusoidal signal similar to the one shown in the left column of Supplementary Fig. 2(a), for which a 100 μs long pure sine with $\omega/2\pi = 87.5$ kHz was generated. As in our experiment a discrete "measurement" value is taken every 2 μs corresponding to a sampling rate of 500 kHz. In order to determine the frequency we guess a pure oscillatory fitting function:

$$N(t) = A\cos(\omega t + \varphi). \tag{S1}$$

The three fitting parameters are the amplitude A, the frequency ω and the phase φ. Since the frequency is not known a priori we instruct the least-squares algorithm to start its search for a minimum in parameter space $(A, \omega, \varphi) = (1, \omega_0, 0)$, where $\omega_0 \in 2\pi \times [20, 200]$ kHz. For each initial value of ω_0 the algorithm converges to some value for the three parameters (A, ω, φ) in the vicinity of the initial parameters (possibly a local minimum, not necessarily the global minimum) and we record the converged $A(\omega)$, see right column of Supplementary Fig. 2(a). The value of ω at which A is maximal (we denote these values $\omega^\star$ and $A^\star$) is the dominant frequency contribution and the *global* minimum in parameter space. As expected for this pure sine, $\omega^\star/2\pi = 87.5$ kHz is obtained. The trustworthiness of the spectrum

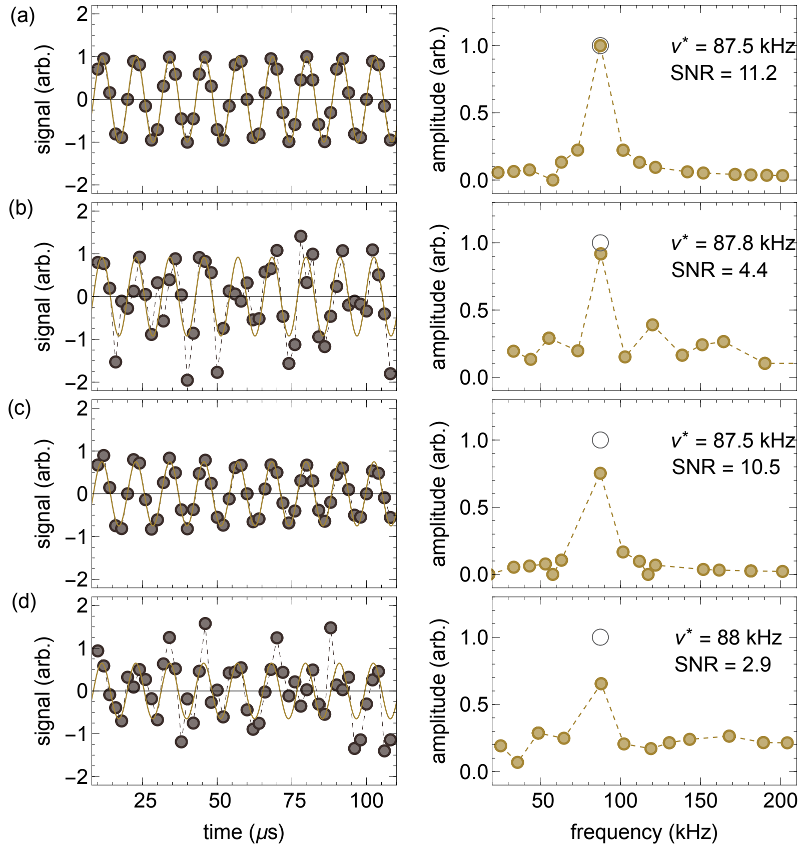

Supplementary Fig. 2. **Three-parameter fit.** The 3PA is applied to four signals to illustrate its working principle. The open circle shows the frequency and amplitude of the pure sine. (a) Pure sine. (b) Noisy sine. (c) Sine with decay. (d) Noisy sine with decay.

is quantified by a signal-to-noise ratio as:

$$\mathrm{SNR} = \frac{A^\star}{\bar{A}}, \tag{S2}$$

where $\bar{A}$ is the mean of all points excluding $A^\star$. For the pure sine, SNR = 11.2. Due to the finite length of the signal, $\bar{A} \neq 0$ and hence the SNR does not diverge as it would for an infinitely long noiseless sine.

We now add white Gaussian noise (WGN) with a standard deviation of 0.5 (half the amplitude) to the pure sine and repeat the procedure in Supplementary Fig. 2(b). Albeit the WGN, the 3PA is able to determine the dominant frequency contribution with an error < 1 kHz (corresponding to the typical errorbar) but with a reduced SNR = 4.4. Note that it is not $A^\star$ that is lowered due to the WGN but $\bar{A}$ which is increased.

The real signal of the DITRIS experiment decays. A decaying sine with characteristic decay time $\tau = 200\ \mu$s is simulated without noise in Supplementary Fig. 2(c). One notes that the obtained $\nu^\star$ is identical to the non-decaying signal of Supplementary Fig. 2(a) although Eq. (S1) was used to determine it, and that the SNR is reduced by less than 10%.

Finally, Supplementary Fig. 2(d) shows a noisy decaying sine representing the real experimental conditions. Despite the fitting function not including the decay and despite the noise, the frequency $\nu^\star$ is found with an error < 1 kHz! Although the SNR is reduced by a factor of ~ 3 with respect to Supplementary Fig. 2(a), the dominant frequency contribution is easy to read off.

B. Alternative analyses

The 3PA is better than a fast Fourier transform (FFT). The main reason for this is the finite sample length. For the signals in Supplementary Fig. 2 the sample length (100 μs) would lead to a frequency resolution of $(100\ \mu\mathrm{s})^{-1} = 10$ kHz (irrelevant of the sampling rate). The accuracy of frequency determination is thus limited to 10 kHz. In our case, where 87.5 kHz is the correct frequency, both the 80 kHz and the 90 kHz point have an amplitude of ~ 0.5, heavily reducing the accuracy and the SNR. The FFT method is very well suited for long samples but not for our relatively short data sets. Nevertheless, see right column of Supplementary Fig. 3, application of a FFT to the experimental signal yields the (approximately) correct dominant frequency distribution.

An alternative to the 3PA is a two-parameter fit analysis (2PA). This involves a fit to Eq. (S1) but using only A and φ as fitting parameters. The frequency is put in by hand and the least-squares algorithm finds the best fitting amplitude and phase for each frequency. Although this method does not suffer from finite resolution, which may be made arbitrarily small, the likelihood analysis, described below, shows its clear disadvantage. In addition, fixing ω does not provide fitting errors for the frequency. The 3PA on the other hand provides a confidence interval for all three parameters.

For completeness and in addition to the 3PA we have analyzed our experimental $N(t)$ with the 2PA and FFT – see Supplementary Fig. 3. Unsurprisingly the same signature is obtained with all three methods.

An illustrative comparison of all three methods (FFT, 2PA and 3PA) can be found in the Supplemental Material of Ref. [1].

C. Likelihood analysis

Here we show that the 3PA is the least likely to be fooled by a false signal. The question we answer here is: For a sample of random numbers, how likely is the 3PA to find a dominant frequency contribution even though none is there (a so-called false-positive)?

To this end we generate 10^4 fake signals, each 100 μs long and sampled at a 500 kHz rate. The random numbers are drawn from a Gaussian random number generator with 0.035 standard deviation (derived from the experimental signals similar to those in Supplementary Fig. 3). We run all three analysis methods on each signal and, as a function of $\mathrm{SNR} = A^\star/\bar{A}$, count the number of false-positives. The result, presented in Supplementary Fig. 4, shows that for $SNR > 1.6$ the 3PA is least likely to be fooled by a false-positive and that for SNR > 2.47 this probability drops below the 10^{-3} level . The other two methods require an SNR of 3.67 (2PA) and 3.59 (FFT) respectively to obtain the same probability.

None of the curves reaches unity for SNR ≤ 1 because we only consider frequency values within our physically accessible window 30 kHz $< \omega^\star/2\pi <$ 120 kHz). Especially the FFT finds mainly high frequencies. The lowest experimental SNR value is indicated by the vertical dashed line in Supplementary Fig. 4. Since the false-positive probability of the 3PA is the lowest in this region we consider it as the most reliable method to analyse the data.

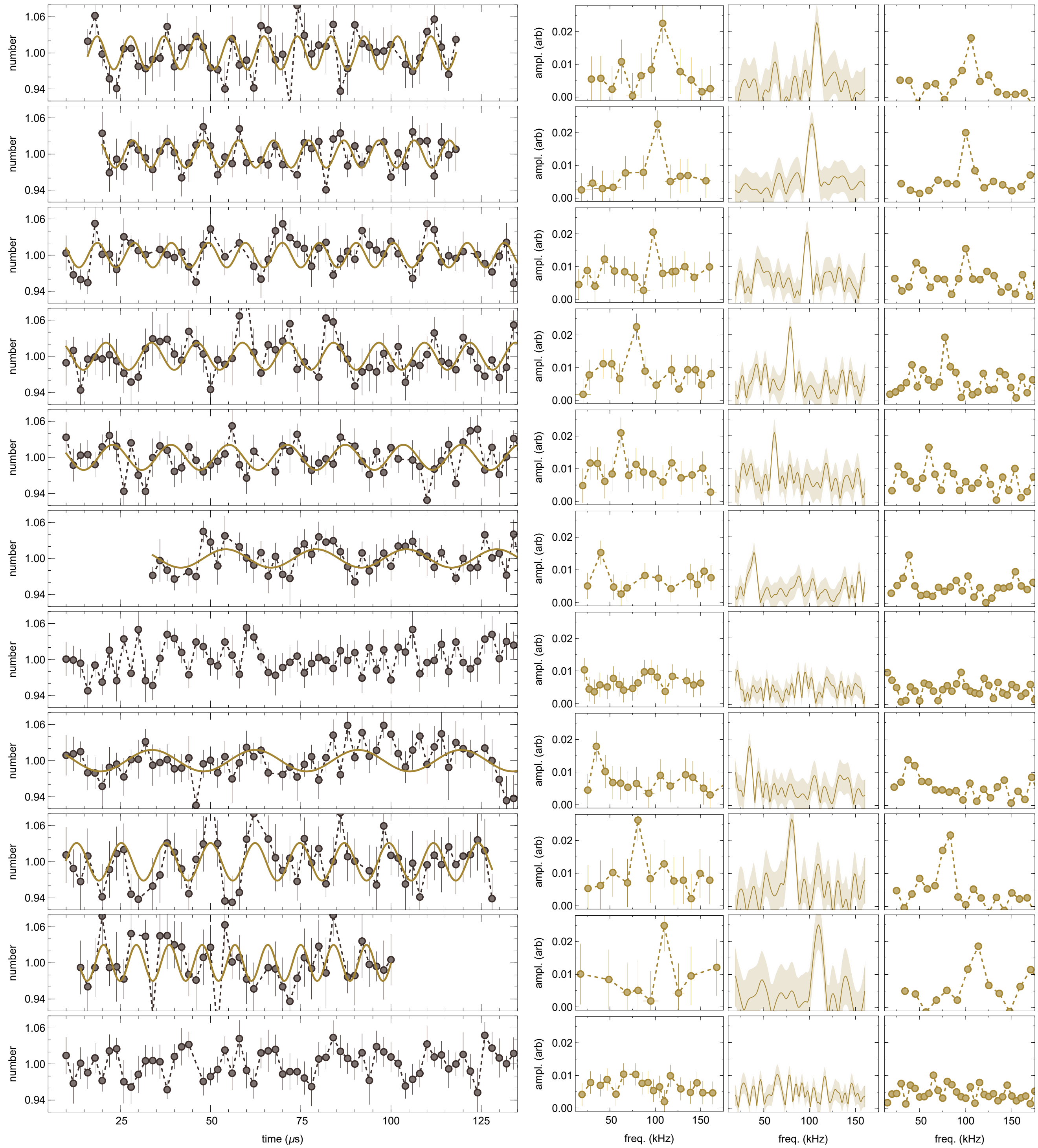

Supplementary Fig. 3. **Measured number of atoms and spectral analysis.** The left column shows the number of atom signal in the double pulse sequence. Each point is the average of 10-20 measurements and the errorbars show the standard deviation. The remaining columns are, from left-to-right, the 3PA, 2PA and FFT of the signals. For the 3PA and 2PA the errorbars and shaded region, respectively, show the 1σ fitting errors. From top-to-bottom the signals were recorded for a scattering length of $a/a_0 = 283$, 265, 248, 197, 185, 176, 164, 160, 157, 156 and 151.

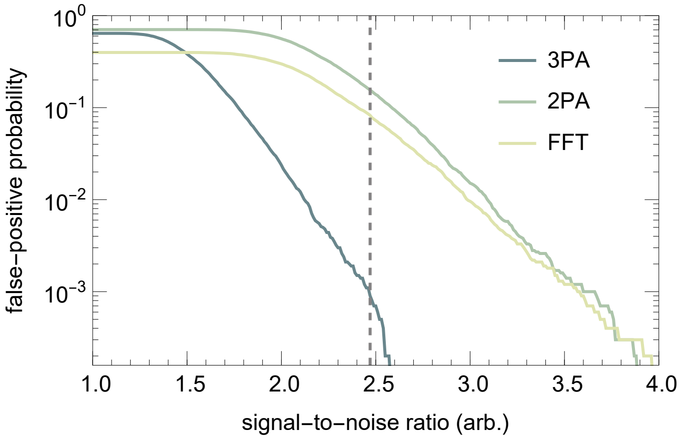

Supplementary Fig. 4. **Likelihood analysis.** The probability of a false-positive is shown as a function of the SNR for the three analysis methods. The vertical dashed line shows the lowest experimental SNR.

Supplementary Note 3. CRAMER-RAO LOWER BOUND

When considering low-SNR sinusoidal data the following question naturally arises: How much could we benefit from increasing the sampling rate at the expense of shortening the sample length? In other words, given a finite number of data points, is it better to spread them out over many oscillations or to sample the first oscillation very densely?

To answer this question we look at the standard signal-processing figure-of-merit called Cramer-Rao lower bound (CRLB) [2]. Consider N samples obtained at times t_n $(n = 1, \dots, N)$:

$$x_n = A\cos(\omega t_n + \varphi) + w_n(0, \sigma), \tag{S3}$$

where w_n is a Gaussian random number with zero mean and standard deviation σ. The values of A, ω and φ are not known. The CRLB provides a mathematical expression for how well their value may be estimated for a given σ and N. In the following we consider two cases:

- $t_n = (n-1)dt$
- $t_n = (n-1)dt/N$

In the first case, increasing N makes the sample longer but the sampling rate $(1/dt)$ remains constant. For the second option the opposite is the case. To find the CRLB we need the probability distribution function (PDF) of the n-th point:

$$p_n(x_n; A, \omega, \varphi) = \frac{1}{\sqrt{2\pi\sigma^2}} \exp\left[-\frac{(x_n - A\sin(\omega t_n + \varphi))^2}{2\sigma^2}\right]. \tag{S4}$$

The PDF of the entire data set $x = \{x_n\}$ is $p(x; A, \omega, \varphi) = \prod_n p_n$. The CRLB theorem claims that the lower bound for estimating A, ω or φ is given by the inverse of the curvature of $p(x; A, \omega, \varphi)$ in parameter space (spanned by A, ω and φ). The curvature, moreover, is given by the negative of the second log derivative. The lower bound is thus computed in two steps. First we must arrange all second partial derivatives of $\ln[p(x; A, \omega, \varphi)]$ into a matrix known as the Fisher information matrix:

$$\hat{F} = \begin{pmatrix} -\frac{\partial^2 \ln p}{\partial A^2} & -\frac{\partial^2 \ln p}{\partial A \partial \omega} & -\frac{\partial^2 \ln p}{\partial A \partial \varphi} \\ -\frac{\partial^2 \ln p}{\partial \omega \partial A} & -\frac{\partial^2 \ln p}{\partial \omega^2} & -\frac{\partial^2 \ln p}{\partial \omega \partial \varphi} \\ -\frac{\partial^2 \ln p}{\partial \varphi \partial A} & -\frac{\partial^2 \ln p}{\partial \varphi \partial \omega} & -\frac{\partial^2 \ln p}{\partial \varphi^2} \end{pmatrix}. \tag{S5}$$

Note that for any element of $\hat{F}$ that depends explicitly on x_n the expectation value weighted by $p(x; A, \omega, \varphi)$ must be taken. In the second step we compute the inverse matrix and keep the on-diagonal elements. The lower bound variance of the i-th parameter estimation is given by $(\hat{F}^{-1})_{ii}$.

We have numerically computed this value as a function of N in both cases outlined above. The frequency lower bound $\text{var}(\omega) \geq (\hat{F}^{-1})_{22}$ is found to be $\sim 1/N^3$ in the first case (increasing N means increasing sample length) and

only $\sim 1/N$ in the second (increasing N means increasing sampling rate). By increasing the sample length one thus benefits from an additional factor of $1/N$ (note that the variance is the square of the standard error). For frequency estimation it is thus advantageous to sample at a low rate and for a long time.

In our experiment the sample length is ultimately limited by the decay of the signal which is $\sim 300\mu$s [1].

Supplementary Note 4. THREE-BODY INTERACTION MODELS FOR NARROW RESONANCES

The major task in solving the three-body problem in the adiabatic hyperspherical representation is to solve the hyperangular adiabatic equation $\hat{H}_{\rm ad}\Phi_\nu(R;\Omega) = U_\nu(R)\Phi_\nu(R;\Omega)$, at fixed values of R, to determine the potentials U_ν and channel functions Φ_ν, both of which are required for the study of the solutions of Eq. (3) in the Methods section. The adiabatic Hamiltonian $\hat{H}_{\rm ad}$,

$$\hat{H}_{\rm ad} = \frac{\hat{\Lambda}^2(\Omega)+15/4}{2\mu R^2}\hbar^2 + \hat{V}_T(R,\Omega) + \hat{H}_{\rm at}, \tag{S6}$$

contains the hyperangular kinetic energy via the hyperangular momentum operator [3], $\hat{\Lambda}$, the internal atomic energies, $\hat{H}_{\rm at}$, as well as all the interatomic interactions of the system,

$$\hat{V}_T(R,\Omega) = \hat{V}(r_{12}) + \hat{V}(r_{23}) + \hat{V}(r_{31}), \tag{S7}$$

where r_{ij} is the distance between atoms i and j, and $\hat{V}$ is the corresponding pairwise interaction.

The solutions of the hyperangular adiabatic equation are obtained by expanding the channel functions Φ_ν on the basis of the separated atomic spins $|\sigma\rangle$

$$\Phi_\nu(R;\Omega) = \sum_\sigma \phi_\nu^\sigma(R;\Omega)|\sigma\rangle. \tag{S8}$$

Applying this expansion to the hyperangular adiabatic equation results in a coupled system of equations for the components of ϕ_ν^σ:

$$\left[\frac{\hat{\Lambda}^2(\Omega)+15/4}{2\mu R^2}\hbar^2 + E_{\rm at}^\sigma - U_\nu(R)\right]\phi_\nu^\sigma(R;\Omega) + \sum_{\sigma'} V_T^{\sigma\sigma'}(R,\Omega)\phi_\nu^{\sigma'}(R;\Omega) = 0, \tag{S9}$$

where $E_{\rm at}^\sigma$ is the sum of the three separated atoms in the $|\sigma\rangle$ spin state.

For our studies on the effect of the resonance width (see Fig. 4 of the main text) we use a simple two-channel model for the interatomic interaction

$$\hat{V}(r) = \begin{pmatrix} v_{\rm bg}(r) & v_{\rm c}(r) \\ v_{\rm c}(r) & v_{\rm bg}(r) \end{pmatrix} \tag{S10}$$

with background interaction, $v_{\rm bg}$, and inter-channel coupling, $v_{\rm c}$, given by

$$v_{\rm bg}(r) = -\frac{C_6}{r^6}\left(1-\frac{\lambda_{\rm bg}^6}{r^6}\right), \tag{S11}$$

$$v_{\rm c}(r) = A_{\rm c}\exp\left[-\frac{(r-r_{\rm c})}{2w_{\rm c}^2}\right]. \tag{S12}$$

In our calculations we adjust $\lambda_{\rm bg}$ to produce a Feshbach resonance with the ^{7}Li background scattering length, $a_{\rm bg} \approx -25a_0$ [4], set $r_{\rm c} = 0$ and $w_{\rm c} = 0.5r_{\rm vdW}$ and vary $A_{\rm c}$ to produce different values for $s_{\rm res}$. We assume the B-field dependent energy difference between open and closed channels to be given by $\epsilon + \delta\mu B$ where we set $\epsilon = 10E_{\rm vdW}$ and $\delta\mu = 6\times10^{-3}E_{\rm vdW}/$G. In Supplementary Fig. 5 we show the effective potentials relevant for Efimov physics at $a = \pm\infty$ and various values of $s_{\rm res}$ between 0.13 and 246, thus covering both the broad and narrow resonance regimes.

For our more quantitative studies of ^{7}Li, the spin states and corresponding separated atomic energies are determined by the hyperfine interactions. We assumed the interatomic interactions to be given in terms of the singlet, $V_{S=0}$, and triplet, $V_{S=1}$, potentials:

$$\hat{V}(r) = \sum_{SM_S} |SM_S\rangle V_S(r)\langle SM_S|. \tag{S13}$$

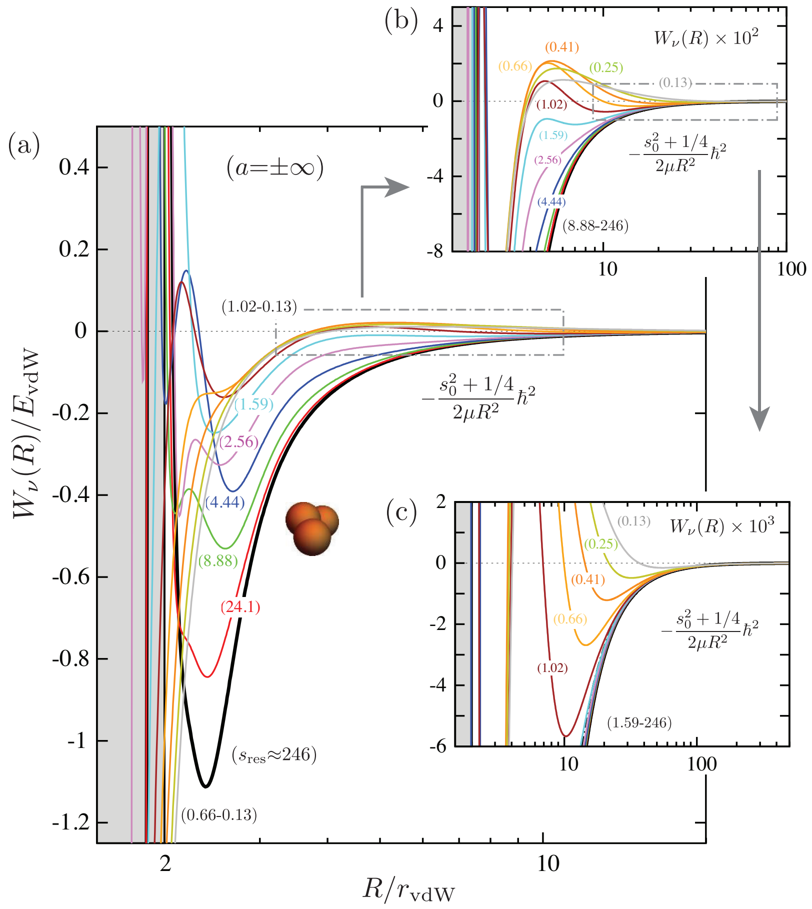

Supplementary Fig. 5. **Reshape of three-body interactions for narrow resonances.** (**a**) Effective potentials, $W(R)$, for the relevant channel supporting an infinity of Efimov states for different values of $s_{\rm res}$ in units of $E_{\rm vdW}=\hbar^2/mr_{\rm vdW}^2$. (**b**) and (**c**) Enhanced views of $W(R)$ illustrating their properties for different values of $s_{\rm res}$: As $s_{\rm res}$ evolves from the regime of broad ($s_{\rm res}\gg 1$) to narrow $s_{\rm res}\ll 1$ resonances a repulsive interaction emerges for $R\gtrsim 4r_{\rm vdW}$ and extending up to $R\approx 3r_*$, where $r_*\approx 1.912r_{\rm vdW}/s_{\rm res}$. The double-well structure of the three-body interaction for narrow resonances allows for trimer states to exist above the atom-dimer continuum for finite values of $a>0$ as shape resonances.

Within our approach, a key approximation is that we introduce a λ_S^6/r^{12} repulsive interaction to the *ab initio* singlet and triplet ^{7}Li Born-Oppenheimer potentials from Ref. [5] in order to restrict the diatomic molecular states to a manageable number (about 100 instead of 1000s) for our three-body calculations. By adjusting the values of λ to produce the correct values for the singlet and triplet scattering lengths [5], this model accurately describes the relevant Feshbach resonances for atoms in the $|F=1,m_F=0\rangle$ hyperfine state. In Supplementary Fig. 6 we show the hyperspherical effective potentials for ^{7}Li demonstrating the existence of the repulsive barrier for both obtained for $a=\pm\infty$ (dashed lines) and $a\approx 5.7r_{\rm vdW}$ (solid lines).

The three-body spin function used in our calculations follows the spectator atom approximation, where two atoms are allowed to interact via spin states satisfying $m_{F_1}+m_{F_2}=0$ while the third atom remains in the $|F_3=1,m_{F_3}=0\rangle$ state. Although this approximation has been shown to be enough to describe the experimental results for ^{39}K [6, 7], this is not the case for ^{7}Li when it comes to reproducing the position of the Efimov resonance in recombination experiments [8]. This result is most likely due to the presence of strong electronic spin exchange for ^{7}Li atoms [9, 10], which would require a larger spin basis to accurately describe the ^{7}Li interactions. Here, in order to set our model to produce results compatible with these observations we introduce a fictitious three-body interaction of the form

$$V_{\rm ex}(R)=-A_{\rm ex}R^{\lambda}{\rm Exp}[-R/\beta], \tag{S14}$$

where we set $\lambda=5$ and $\beta=0.2r_{\rm vdW}$ and tune $A_{\rm ex}$ to fit the position of the $a<0$ Efimov resonance of Ref. [8]. While this approach leads to an atom-dimer Efimov resonance for $a>0$ with energies comparable to those observed here for ^{7}Li, the calculated lifetimes are on the order of 10s of μs. For our simulations shown in Fig. 5 of the main text, we

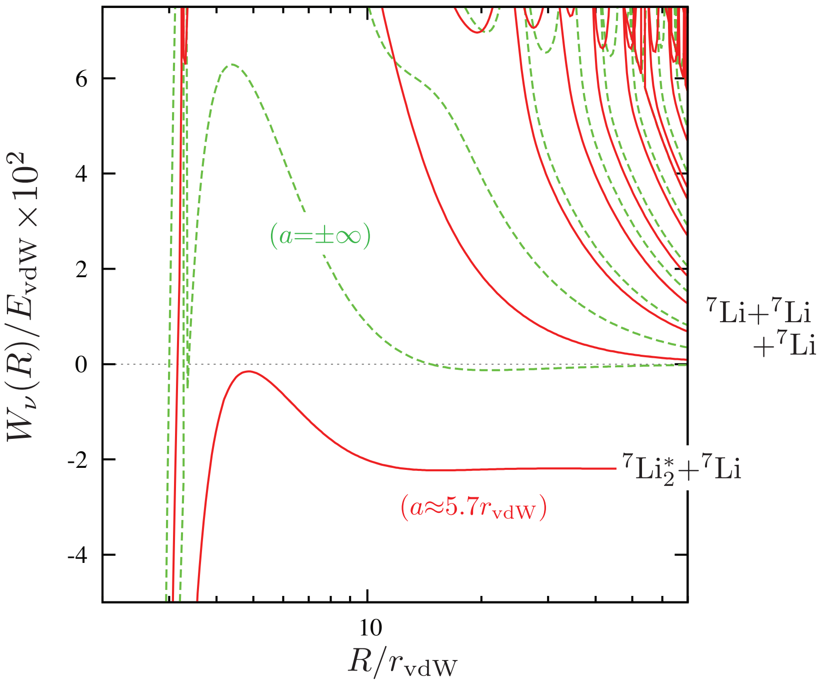

Supplementary Fig. 6. **Effective potentials for ^{7}Li atoms,** in van der Waals units of length, $r_{\rm vdW}$, and energy, $E_{\rm vdW}=\hbar^2/mr_{\rm vdW}^2$. Potentials where $W(R)>0$ for $R\gg r_{\rm vdW}$ correspond to three-body continuum channels while potentials where $W(R)<0$ for $R\gg r_{\rm vdW}$ describe atom-dimer channels. Dashed and solid lines represent the potentials for $a=\pm\infty$ and $a\approx 5.7r_{\rm vdW}$, respectively.

have turned off non-adiabatic coupling between deeply-bound molecular states in order to set a lifetime comparable to the experiments.

SUPPLEMENTARY REFERENCES

[1] Yudkin, Y., Elbaz, R., Giannakeas, P., Greene, C. H. & Khaykovich, L. Coherent superposition of Feshbach dimers and Efimov trimers. *Phys. Rev. Lett.* **122**, 200402 (2019).

[2] Kay, S. M. *Fundamentals of Statistical Signal Processing: Estimation Theory* (Prentice Hall PTR, Upper Saddle River, New Jersey, 1993).

[3] Suno, H., Esry, B. D., Greene, C. H. & Burke, J. P. Three-body recombination of cold helium atoms. *Phys. Rev. A* **65**, 042725 (2002).

[4] Pollack, S. E. *et al.* Extreme tunability of interactions in a ^{7}Li Bose-Einstein condensate. *Phys. Rev. Lett.* **102**, 090402 (2009).

[5] Julienne, P. S. & Hutson, J. M. Contrasting the wide Feshbach resonances in ^{6}Li and ^{7}Li. *Phys. Rev. A* **89**, 052715 (2014). URL https://link.aps.org/doi/10.1103/PhysRevA.89.052715.

[6] Chapurin, R. *et al.* Precision test of the limits to universality in few-body physics. *Phys. Rev. Lett.* **123**, 233402 (2019).

[7] Xie, X. *et al.* Observation of Efimov universality across a non-universal Feshbach resonance in ^{39}K. *Phys. Rev. Lett.* **125**, 243401 (2020).

[8] Gross, N., Shotan, Z., Kokkelmans, S. & Khaykovich, L. Nuclear-spin-independent short-range three-body physics in ultracold atoms. *Phys. Rev. Lett.* **105**, 103203 (2010).

[9] Li, J.-L., Secker, T., Mestrom, P. M. A. & Kokkelmans, S. J. J. M. F. Strong spin-exchange recombination of three weakly interacting ^{7}Li atoms. *Phys. Rev. Res.* **4**, 023103 (2022). URL https://link.aps.org/doi/10.1103/PhysRevResearch.4.023103.

[10] van de Kraats, J., Ahmed-Braun, D. J. M., Li, J.-L. & Kokkelmans, S. J. J. M. F. Emergent inflation of the Efimov spectrum under three-body spin-exchange interactions. *arXiv:2309.13128* (2023).
